# A Tribute to English Medical Science

**Published:** 1920-06-19

**Authors:** 


### THE ROCKEFELLER GIFT.

### A Tribute to English Medical Science.

Clearly to appreciate the full meaning of the gift of £1,205,000 offered by the Rockefeller Foundation to University College Hospital Medical School and University College the workings of this American fund must be understood in some detail.

In the first place, the avowed purpose of the foundation is to promote the well-being of mankind throughout the whole world and it is left to the trustees to select the institutions through whose agency this magnificent object may be accomplished. In this instance their choice has fallen upon a recently developed section in medical teaching the principles of which have been frequently advocated upon both sides of the Atlantic and which is certainly calculated both directly and indirectly to aid the progress of medical research. It is true, however, that the colleges thus fortunately selected are not alone in adopting this particular method of training the medical generations of the future, although we believe it can with justice be said that the authorities of University College are extremely well advanced with their plans of organisation and we know them to be fortunate possessors of some of the most brilliant medical teachers in the country.

In summary, the academic principles which have received this valuable encouragement provide the best perfection of the "unit" system. Units are provided in connection with surgery, medicine, and obstetrics, and each is under the personal direction of a professor who will give practically his whole time to educational and research work. His staff of assistants, who are, of course, highly qualified men and not necessarily "juniors," will act some in a full-time, some in a part-time capacity and in this way will be obtained an ideal blending of the institutional and practitioners' insight to the various aspects of medical problems. The master hand in the unit will give his undivided attention to the work and be in a position to call upon such phases of outside experience as are from time to time necessary, and since 50 to 100 beds and a special out-patient department are the special property of the unit, together with excellent laboratory and *post-mortem* services, the completeness of scientific opportunity is obvious.

In the original discussions the scheme as a whole was summarised by the Rockefeller Executive Committee as follows: (1) an institute of anatomy; (2) increase of clinical facilities; (3) clinical laboratories planned; (4) increased maintenance costs; (5) closely unified administration; and after completely surveying the situation in London they expressed the view that to help such a scheme of development would be in full accord with the purpose of the foundation. Their desire was, however, to help truly to carry out such development rather than to relieve any existing expenditure, and as a final result of the negotiations the University College authorities were able to state that the Rockefeller Foundation offered to give to University College Hospital Medical School the sum of £400,000 towards a building and equipment programme substantially as submitted by the authorities of the schools, and the further sum of £435,000 towards the support of clinical facilities and teaching in substantial conformity at the outset with the proposed plan, making a total of £835,000 for the Medical School. To the University of London in behalf of the University College was offered the sum of £190,000 towards a building and equipment programme, substantially as submitted by the authorities of the College, and the further sum of £180,000 towards endowment of laboratory teaching in substantial conformity at the outset with the proposed plan, making a total of £370,000 for the College and a grand total of £1,205,000. This sum is probably the largest single gift which has ever been made for educational purposes in this country, and the admirable spirit with which it has been given, and the complete absence of dictation on the part of the Executive Committee of the Foundation, make it a more than memorable event in the development of a gradually tightening bond of sympathy between the professions of the two great English-speaking nations. On the other hand, we must realise its true relation to the present financial crisis among the hospitals of Great Britain.

As *The Times* pointed out, this event essentially amounts to an extraordinarily liberal and whole-hearted support given on the part of American circles to a great experiment in medical education. True it is an experiment in the success of which we have every confidence and which should blend happily those various teaching requirements which we have repeatedly urged in these pages; but at the same time, it must be remembered that practically every school and every hospital in Great Britain is in urgent need of funds at the present time. The future may hold for University College and Hospital a brilliant academic future, and a magnificent example may be shown of what could be accomplished if similar financial support were forthcoming for all the very necessary institutional developments which the economic situation of to-day demands. But those who can give or can influence the giving of money towards alleviation of the straits in which British hospitals generally find themselves to-day, must realise that the gift of the Rockefeller Foundation is of an educational rather than institutional value. It aims to alleviate the sufferings of mankind through the highest available channels, namely, medical research and the better education of the practitioner. The immediate needs of the sick, the sadly deficient hospital accommodation, and the extreme difficulties under which the greater part of the institutional work is nowadays carried on, are problems with us still.

As we go to press there is still some uncertainty as to the exact relation between the conditions of this gift and the suggested further appeal for maintenance funds to be made by the hospital concerned, and under these circumstances we reserve our judgment on the matter.

